# Cinacalcet Perturbs Membrane Permeability of bEND.3 Endothelial Cells and Suppresses Cell Proliferation

**DOI:** 10.33549/physiolres.935632

**Published:** 2025-10-01

**Authors:** Yu-Jen CHEN, Chen-Hsiu LIN, Cheng-An WANG, Paul CHAN, Cing Yu CHEN, Yu-Wen WANG, Lian-Ru SHIAO, Yuk-Man LEUNG, Jong-Shiuan YEH

**Affiliations:** 1 Division of Cardiovascular Medicine, Department of Internal Medicine, Wan Fang Hospital, Taipei Medical University, Taipei, Taiwan; 2 Division of Cardiology, Department of Internal Medicine, School of Medicine, College of Medicine, Taipei Medical University, Taipei, Taiwan; 3 Taipei Heart Institute, Taipei Medical University, Taipei, Taiwan; 4 Institute of Public Health, National Yang Ming Chiao Tung University, Taipei, Taiwan; 5 Graduate Institute of Clinical Medicine, College of Medicine, Taipei Medical University, Taipei, Taiwan; 6 Department of Cosmetic Science, Providence University, Taichung, Taiwan; 7 School of Pharmacy, China Medical University, Taichung, Taiwan; 8 Department of Biotechnology and Pharmaceutical Technology, Yuanpei University of Medical Technology, Hsinchu, Taiwan; 9 Department of Physiology, China Medical University, Taichung, Taiwan

**Keywords:** Ca^2+^-sensing receptors, Endothelium, Cinacalcet, Ca^2+^, Membrane permeability

## Abstract

Ca^2+^-sensing receptors (CaSR) are G-protein coupled receptors activated by elevated concentrations of extracellular Ca^2+^. Cinacalcet, a positive allosteric CaSR modulator, is used as a calcimimetic to inhibit parathyroid hormone release and thus lower serum Ca^2+^ in hypercalcemic patients. There is a recent trend of repurposing cinacalcet in multiple applications such as anti-cancer actions, treatment of diarrhea and prevention of kidney cyst formation. In this study we investigated whether cinacalcet inhibited proliferation of endothelial cells (EC). Inhibition of EC proliferation offers an anti-angiogenesis mechanism in suppressing tumor growth. Cinacalcet at ≥18 μM caused mitochondrial membrane depolarization, suppressed proliferation and induced apoptosis in mouse bEND.3 EC. In 2 mM Ca^2+^-containing bath solution, cinacalcet (≥18 μM) caused an initial rise in [Ca^2+^]_i_ followed by fura-2 leakage. Similar results were obtained in Ca^2+^-free or 4 mM Ca^2+^-containing bath solution. Cinacalcet-elicited Ca^2+^ signal was unaffected by NPS 2143, a negative allosteric modulator of CaSR. Cinacalcet also increased Ni^2+^ leakage and trypan blue uptake into cells. Cinacalcet-induced membrane leakiness and cytotoxicity did not appear to be related to membrane fluidity changes. These data suggest cinacalcet, in a manner independent of CaSR stimulation and membrane fluidity perturbation, caused membrane leakiness, eventually leading to inhibition of EC proliferation and EC death.

## Introduction

Ca^2+^-sensing receptors (CaSR) are G-protein-coupled receptors activated by elevated extracellular Ca^2+^ concentration, amino acid and polyamines [1]. CaSR is ubiquitously expressed in many tissues and cell types. Amongst many functions, CaSR is involved in regulation of parathyroid hormone release, neuritogenesis and synaptic transmission in neurons, control of vascular tone *via* endothelial cell (EC) ion channels, and regulation of ion transport in kidney epithelial cells [2,3]. CaSR has been demonstrated to activate a number of signaling pathways, namely, G_q/11_, G_12/13_, Gi and Gs and ERK_1/2_ [4–9].

Cinacalcet is a positive allosteric modulator (PAM) of CaSR, acting by enhancing the latter’s affinity to extracellular Ca^2+^. Cinacalcet is therefore a calcimimetic agent which could be used to treat parathyroid carcinoma, patients having secondary hyperparathyroidism and receiving dialysis, and hyper-calcemia in patients with primary hyperparathyroidism [10]. A recent trend of repurposing cinacalcet has been emerging in treatments such as suppression of CFTR-mediated secretory diarrhea [11], prevention of kidney cyst formation [12] and bactericidal effects towards multidrug-resistant strains [13–15]. Cinacalcet has also been deployed as an anti-cancer agent. For instance, cinacalcet was used to suppress Yes-associated protein expression to arrest proliferation of hepatocellular carcinoma [16]. Cinacalcet inhibits neuroblastoma tumor growth [17] and growth of human lung adenocarcinoma [18].

During tumor growth, new blood vessel formation is needed to supply oxygen and nutrients. Thus, EC proliferate and migrate from the already-established vasculature to form new blood vessels [19]. Tumor-associated ECs are highly proliferative, and possess the ability to initiate immunosuppressive mechanisms [20]. It would be of great interest to identify drug candidates to inhibit EC proliferation. In this study we observed that cinacalcet caused membrane leakiness in mouse microvascular bEND.3 EC in a manner independent of CaSR activation and membrane fluidity changes, resulting in inhibition of cell proliferation and apoptotic death.

## Materials and Methods

### Materials and cell culture

Fura-2 AM was purchased from Calbiochem-Millipore. Cinacalcet and NPS 2143 were from Tocris (Bristol, U.K.). Carbonyl cyanide-p-trifluoromethoxyphenylhydrazone (FCCP), trypan blue and ethanol were from Sigma-Aldrich (St. Louis, MO, USA). Brain microvascular bEND.3 cells were cultured in Dulbecco’s modified Eagle’s medium (DMEM) supplemented with 1 % penicillin/streptomycin and 10 % fetal bovine serum (Invitrogen).

### Assay of cell viability

bEND.3 cells were cultured in 96-well plates at a density of 1.0×10^4^/well for 2 days to reach approximately 80 % confluence. Subsequently the cells were treated with different agents for 2.5 h. 3-(4,5-di-methylthiazol-2-yl)-2,5-diphenltetrazolium bromide (MTT; final concentration of 0.5 mg/ml) was then added to each well and further incubated for a duration of 4 h. Culture medium was discarded and DMSO (100 μl) was then added per well for another 15 min with mild shaking to let precipitates dissolve. Absorbance at 595 nm was detected using an ELISA reader; absorbance was used to indicate viability of cells or metabolic activities. A reduction in MTT absorbance indicates death of cells, reduction of cell proliferation or metabolic activity loss.

The trypan blue exclusion method was also used to quantitate the number of viable cells. Cells were initially seeded in 12-well plates at a density of 3×10^4^ cells/well, treated with DMSO or various agents and then incubated for 2 days. Viable cells, which were unstained by trypan blue (final concentration=0.4 %), were counted using a hemocytometer.

In another set of experiments, trypan blue uptake, as an indicator of loss of membrane integrity, was measured spectrophotometrically. bEND.3 cells were cultured in 96-well plates at a density of 1.0×10^4^/well for 2 days to reach approximately 80 % confluence. The cells were treated with different agents for various time periods and subsequently trypan blue (final concentration=0.4 %) was added to the cells for 5 min. After washing, absorbance at 595 nm was detected using an ELISA reader (Biotek ELx808). An increase in absorbance indicates uptake of trypan blue and thus loss of membrane integrity.

### Microfluorimetric measurement of cytosolic Ca^2+^ changes and fura-2 leakage

Microfluorimetric measurement of cytosolic Ca^2+^ changes was conducted using fura-2 as Ca^2+^-sensitive probe [21,22]. In brief, the cells were grown on small glass cover slips, and incubated with 5 μM fura-2 AM (Invitrogen, Carlsbad, CA) for 1 h at 37 °C. The cells were then washed in bath solution, containing (mM): 140 NaCl, 4 KCl, 1 MgCl_2_, 2 CaCl_2_, 10 HEPES (pH 7.4 adjusted with NaOH). Ca^2+^-free solution was the same as the bath solution described above except that Ca^2+^ was absent and 100 μM EGTA was added. Cells were alternately excited with 380 nm and 340 nm (switching frequency 1 Hz) with the aid of an optical filter changer (Lambda 10-2, Sutter Instruments). Emission wavelength was set at 500 nm and images were acquired with a CCD camera (CoolSnap HQ2, Photometrics, Tucson, AZ, USA) connected to an inverted Nikon TE2000-U microscope. When fura-2 leakage was measured, excitation wavelength was 360 nm and emission wavelength was 500 nm. Data were analyzed by an MAG Biosystems Software (Sante Fe, MN, USA). Experiments were performed at room temperature (~25 °C).

### Assay of divalent cation influx

Cells were seeded in a 96-well plate at a density of 1×10^4^ cells/well and incubated for 2 days. Cells were loaded with 5 μM fura-2 AM (Invitrogen, Carlsbad, CA) for 1 h at 37 °C. To assay divalent cation influx, cells were washed and treated with DMSO or 30 μM cinacalcet for 20 min. Thereafter the supernatants were removed and bath solutions without or with 1 mM NiCl_2_ or CoCl_2_ were added for 10 min. Fluorescent emission was measured by a Varioskan LUX multimode microplate reader (Thermo Fisher Scientific, Waltham, MA, USA). Excitation and emission wavelengths were set at 360 nm and 500 nm. Since Ni^2+^ and Co^2+^ quench fura-2 fluorescence, a reduction of fluorescence indicated influx of these ions.

### Assay of caspase-9

Caspase-9 level was measured using an ELISA kit (Cat. #E-EL-H0663; Elabscience, Houston, TX, USA) following the instructions in the manufacturer’s manual.

### Measurement of mitochondrial membrane potential

Mitochondrial membrane potential was quantified by a Mitochondrial Membrane Potential Assay Kit (#12664; Cell Signaling, Danvers, MA, USA) [23]. The cells were seeded at a density of 5×10^4^ cells per well to settle overnight. The cells were treated with DMSO or other agents for 24 h. 2 μM JC-1 was then added to each well for 30 min. Fluorescent emission was measured by a Varioskan LUX multimode microplate reader (Thermo Fisher Scientific, Waltham, MA, USA). Excitation wavelength was at 485 nm and dual emission wavelengths were at 520 and 590 nm. Mitochondrial membrane potential was quantitated as the ratio RFU of red emission (590 nm)/RFU of green emission (520 nm).

### Measurement of membrane fluidity

Membrane fluidity was measured by a membrane fluidity assay kit (ab189819; Abcam, Cambridge, U.K.) according to the manufacturer’s manual. Briefly, the cells were seeded at a density of 1×10^4^ cells per well for 2 days. After the cells were incubated in the presence of fluorescent lipid reagent for 1 h, they were washed and then treated with DMSO, cinacalcet or ethanol for different time periods. Fluorescent emission was measured by a Varioskan LUX multimode microplate reader (Thermo Fisher Scientific, Waltham, MA, USA). Excitation wavelength was at 350 nm and dual emission wavelengths were at 400 and 470 nm. Membrane fluidity was quantitated as the ratio of emission at 470 nm to emission at 400 nm.

### Statistical analysis

Data are means ± SEM. Unpaired or paired Student *t*-test was used where appropriate to compare 2 groups. Multiple groups were analyzed by ANOVA, followed by the Tukey’s HSD *post hoc* test. *p*<0.05 was considered statistically significant.

## Results

**Fig. 1:**
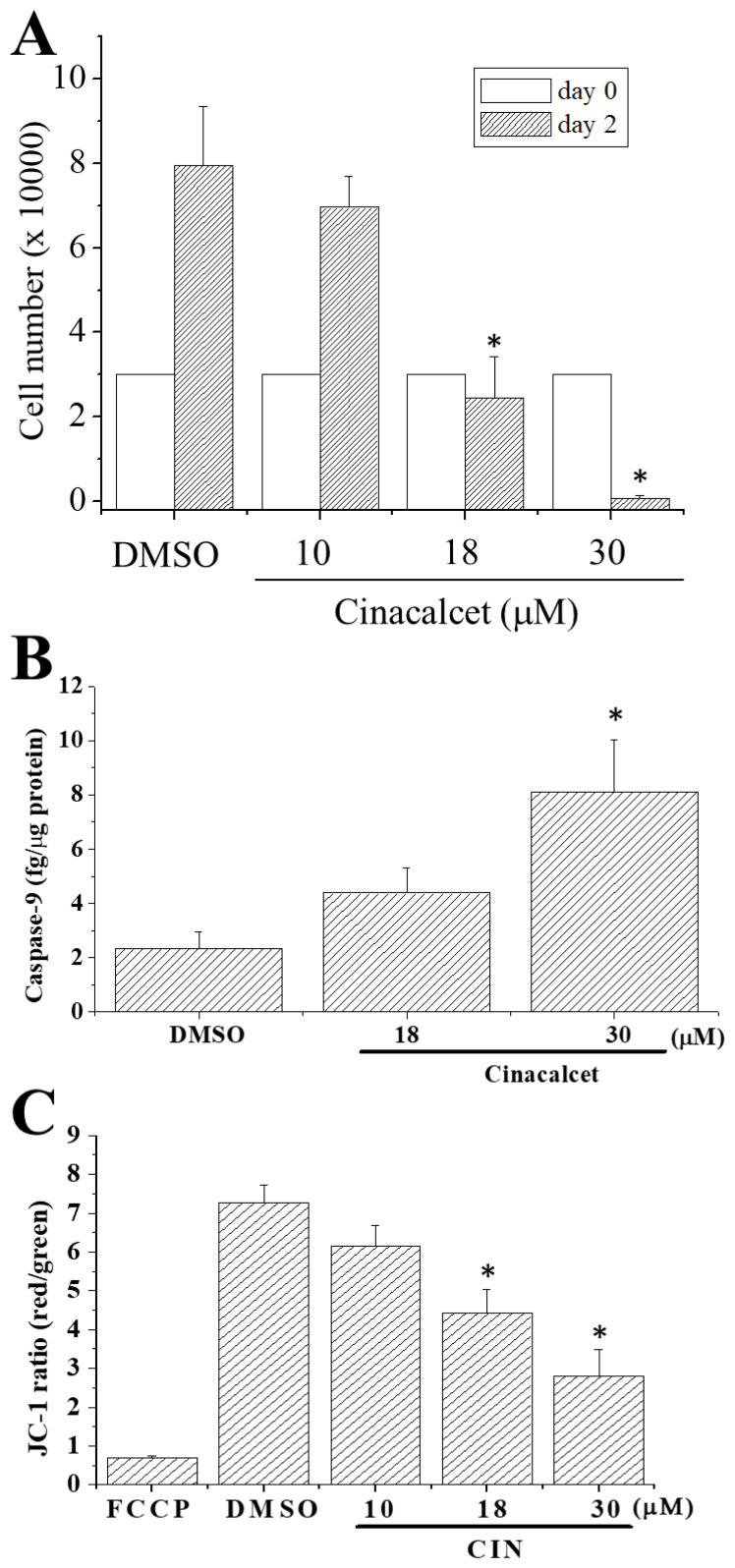
Effects of cinacalcet on cell viability, caspase-9 generation and mitochondrial membrane potential. (**A**) bEND.3 cells were seeded in 24-well plates at an initial concentration of 3×10^4^ cells/well. They were then treated with DMSO or different concentrations of cinacalcet for 2 days before the trypan blue exclusion test was performed to quantify the number of viable cells. (**B**) bEND.3 cells were treated with DMSO, 18 or 30 μM cinacalcet for 36 h and caspase-9 was assayed. (**C**) Cells were treated with DMSO or different concentrations of cinacalcet for 36 h and mitochondrial membrane potential was measured as described in the Methods section. FCCP (4-h treatment) was used as a positive control for membrane potential collapse. Results are mean ± SEM from 4–5 separate experiments. * significantly (*p*<0.05) different from the DMSO control group.

Cinacalcet reportedly ameliorates endothelial dysfunction in patients with secondary hyperparathyroidism [24], but its effect on viability and functions of normal endothelial cells is unclear. We first examined whether treatment of bEND.3 cells with cinacalcet would affect cell proliferation or cause cell death. As shown in Figure 1A, cinacalcet at 18 μM ceased cell growth whilst at 30 μM caused almost complete cell death after two days of culture. At 30 μM, cinacalcet increased the level of caspase-9, a marker of apoptosis, suggesting that cinacalcet caused apoptotic cell death (Fig. 1B). The effect of cinacalcet on mitochondrial membrane potential was examined (Fig. 1C). At 18 and 30 μM, cinacalcet caused significant depolarization of mitochondrial membrane potential; FCCP was used as a positive control to cause mitochondrial potential collapse.

**Fig. 2:**
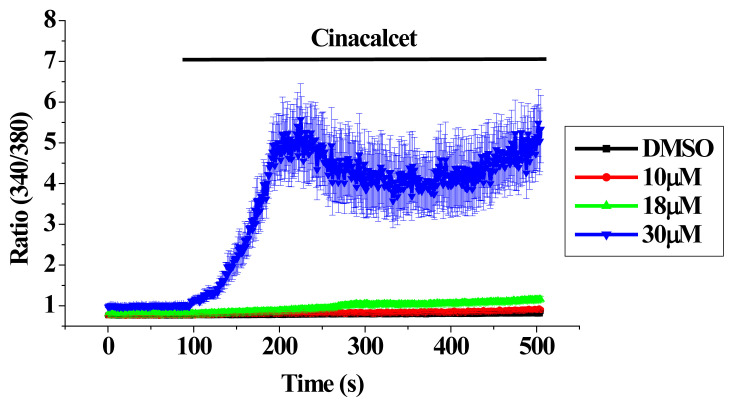
Effect of cinacalcet on cytosolic Ca^2+^ concentration changes. Cells were bathed in 2 mM Ca^2+^-containing solution and treated with different concentrations of cinacalcet. Results are mean ± SEM; individual group had 21–26 cells from 3 separate experiments.

**Fig. 3:**
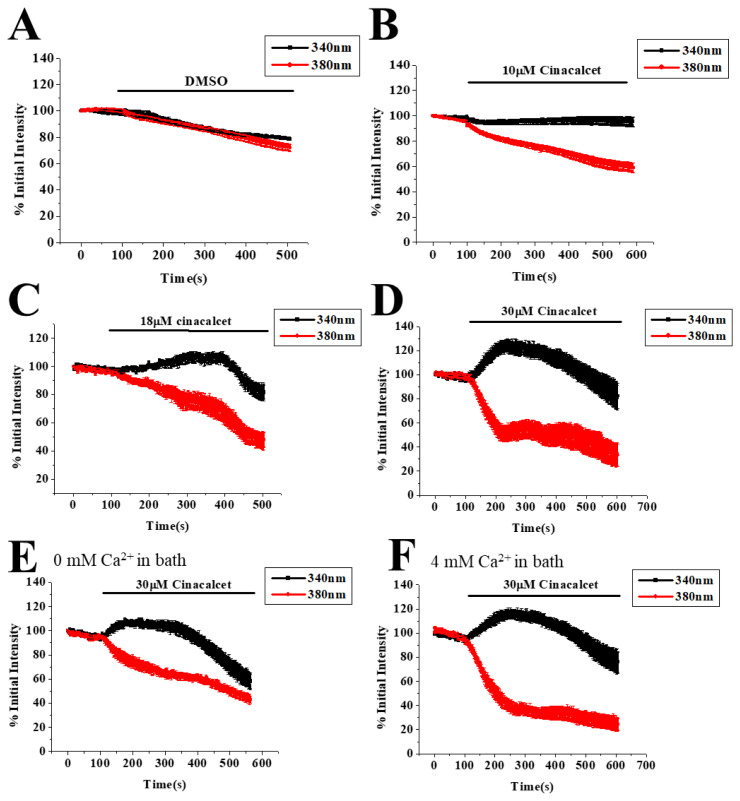
Effect of cinacalcet on cytosolic changes in fura-2 fluorescence at 340 nm and 380 nm excitation. (**A**) to (**D**) Cells were bathed in 2 mM Ca^2+^-containing solution and treated with DMSO or different concentrations of cinacalcet. Cells bathed in Ca^2+^-free solution (**E**) or in 4 mM Ca^2+^-containing solution (**F**) were treated with 30 μM cinacalcet. Results are mean ± SEM; individual group had 21–26 cells from 3 separate experiments.

We next examined the effects of cinacalcet on [Ca^2+^]_i_. As shown in Figure 2, addition of DMSO to bEND.3 cells did not elevate [Ca^2+^]_i_; cinacalcet treatment concentration-dependently raised [Ca^2+^]_i_. The Ca^2+^ responses triggered by 30 μM cinacalcet were disproportionately large and variable. We hence analyzed fluorescence changes at 340 and 380 nm excitation (Fig. 3). DMSO did not cause significant changes in fluorescence at 340 and 380 nm excitation (note that the mild decline in fluorescence at both wavelengths was due to inevitable photobleaching with time). As shown in Fig. 3B, treatment with 10 μM cinacalcet elicited mild increase and decrease in fluorescence at 340 and 380 nm excitation, respectively, therefore indicating a rise in [Ca^2+^]_i_. Addition of a higher concentration (18 μM) of cinacalcet caused in the first 5 min an increase and decrease in fluorescence at 340 and 380 nm excitation, respectively (thus a rise in [Ca^2+^]_i_); thereafter a decrease in fluorescence at both 340 and 380 nm excitation was observed, suggesting it was not [Ca^2+^]_i_ elevation but possibly fura-2 leakage (Fig. 3C; also Fig. 5A). Similar finding was obtained with 30 μM cinacalcet, with the initial rise and fall in fluorescence at 340 and 380 nm excitation, respectively, occurring at a faster rate; the fall in fluorescence at 340 and 380 nm excitation occurred earlier (Fig. 3D). Addition of 30 μM cinacalcet in Ca^2+^-free solution yielded similar results albeit with weaker changes in magnitude (Fig. 3E). In 4 mM Ca^2+^-containing bath solution (Fig. 3F), 30 μM cinacalcet yielded similar pattern of changes.

**Fig. 4:**
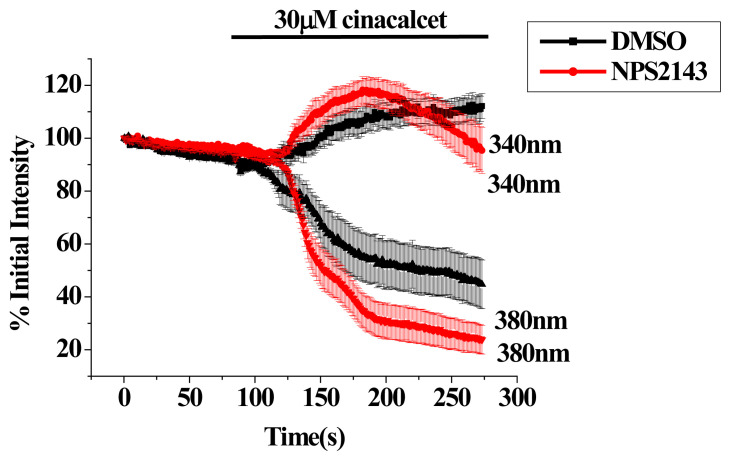
Cinacalcet-elicited cytosolic Ca^2+^ changes were independent of CaSR. Fura-2 fluorescence changes at 340 nm and 380 nm excitation were measured in bEND.3 cells. Cells were bathed in 2 mM Ca^2+^-containing solution and pre-treated with DMSO or 3 μM NPS 2143. Cells were then stimulated with 30 μM cinacalcet. Results are mean ± SEM; each group had 16–24 cells from 3 separate experiments.

To examine if the initial Ca^2+^ signal (indicated as opposite changes in fluorescence at 340 and 380 nm excitation) was caused by cinacalcet *via* CaSR activation, we examined the effect of NPS 2143, a negative allosteric modulator of CaSR; we have shown in our previous report that NPS 2143 inhibited Ca^2+^ signal caused by high Ca^2+^ in bEND.3 cells [25]. As shown in Figure 4, cinacalcet-elicited Ca^2+^ signal was not affected by NPS 2143, suggesting the Ca^2+^ signal caused by 30 μM cinacalcet was not due to CaSR activation.

**Fig.5:**
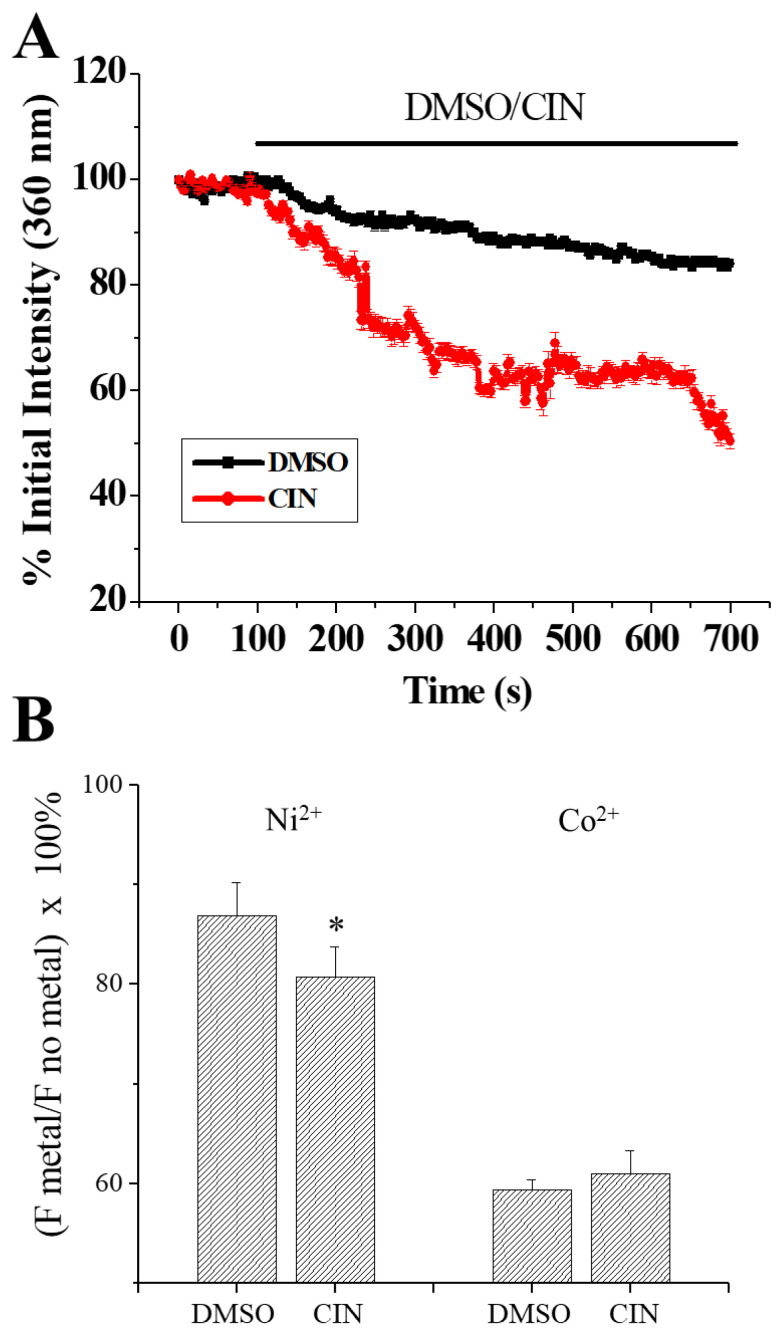
Cinacalcet caused leakage of fura-2 and Ni^2+^. (**A**) Fura-2 fluorescence changes at 360 nm excitation were measured in bEND.3 cells. Cells were bathed in 2 mM Ca^2+^-containing solution and treated with DMSO or 30 μM cinacalcet. Significant difference (p<0.05) between DMSO group and cinacalcet group began at 132 s. Results are mean ± SEM; individual group had 20–41 cells from 3 separate experiments. (**B**) Cells were treated with DMSO or 30 μM cinacalcet for 20 min, supernatants were then removed and bath solutions without or with 1 mM NiCl_2_ or CoCl_2_ were added for 10 min. The extent of divalent cation influx was quantified by (fluorescence in the presence of divalent metal/fluorescence in the absence of divalent metal) × 100 %. Results are mean ± SEM from 3 separate experiments. * significantly (*p*<0.05) different from the DMSO control group.

To verify whether cinacalcet caused fura-2 leakage, we performed the experiment with an excitation wavelength at 360 nm, a wavelength at which fura-2 fluorescence is not affected by Ca^2+^ concentrations. As shown in Figure 5A, cinacalcet caused a gradual drop in fluorescence suggesting leakage of fura-2.

Since cinacalcet caused fura-2 leakage, we proceeded to examine if cinacalcet enhanced membrane leakiness to Ni^2+^ and Co^2+^, two divalent cations which are often used as non-selective Ca^2+^ channel blockers. Since Ni^2+^ and Co^2+^ quench fura-2 fluorescence, a reduction of fluorescence indicated influx of these ions. As shown in Figure 5B, the 12 % reduction in fluorescence in Ni^2+^-added DMSO-treated group suggest a basal Ni^2+^ leak; cinacalcet treatment caused a small but significant augmentation of Ni^2+^ leak. There was a 40 % reduction in fluorescence in Co^2+^-added DMSO-treated group, suggesting a basal Co^2+^ leak; by contrast, cinacalcet treatment did not enhance Co^2+^ leak.

**Fig. 6:**
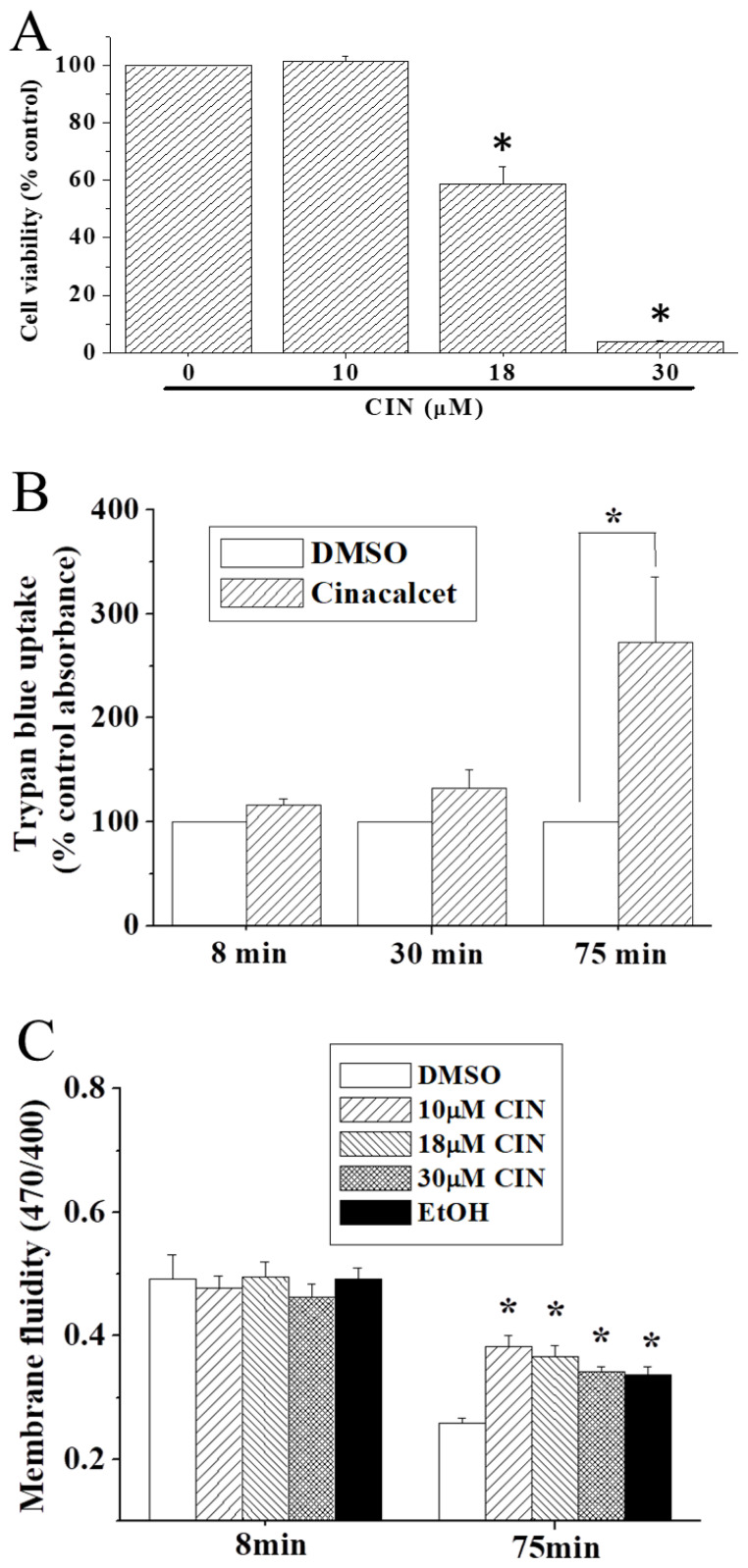
Cinacalcet’s effects on cell viability, trypan blue uptake and membrane fluidity. (**A**) Cells were treated with DMSO or different concentrations of cinacalcet for 2.5 h before subject to MTT assay. (**B**) Cells were treated with DMSO or 18 μM cinacalcet for various time periods. Measurement of absorbance of trypan blue-stained cells was performed as described in the Methods. (**C**) Cells were treated with DMSO, different concentrations of cinacalcet or 2 % ethanol for different time periods before subject to membrane fluidity assay. Results are mean ± SEM from 4 separate experiments. * significantly (*p*<0.05) different from the DMSO control group.

We asked if cinacalcet-induced membrane leakiness would result in a rapid loss of cell viability. Results in Figure 6A indicated that at 2.5 h, cinacalcet (≥18 μM) already caused a drastic decrease in cell viability. Cinacalcet-induced loss of membrane integrity was also assayed by trypan blue uptake measured spectrophotometrically. As shown in Figure 6B, cinacalcet (18 μM) treatment significantly increased trypan blue uptake after 75 min. We also asked if cinacalcet increased membrane permeability by affecting membrane fluidity. Thus, we examined the effects of 2 % ethanol (as positive control, [26]) and cinacalcet on membrane fluidity. Results indicated that cinacalcet or ethanol did not affect membrane fluidity at 8 min, but caused a moderate increase in membrane fluidity at 75 min (Fig. 6C).

## Discussion

The affinity of cinacalcet for heterologously expressed CaSR is in the low micromolar range (see [3] for a review and references therein). In our previous study, we have shown protein (western blot) and functional (activation by millimolar levels of Ca^2+^, Sr^2+^ and La^3+^) expression of CaSR in bEND.3 EC [25]. In this work, cinacalcet at 10 μM only elicited a mild Ca^2+^ response in bEND.3 cells (Fig. 2). A higher concentration (30 μM) of cinacalcet caused a rapid and much larger elevation of cytosolic Ca^2+^ (Fig. 2); the latter however, could not be prevented by NPS2143 (negative allosteric modulator of CaSR) (Figs 3 and 4), suggesting an independence of CaSR activation. As a PAM, cinacalcet action requires the presence of extracellular Ca^2+^: cinacalcet enhances CaSR sensitivity to Ca^2+^ [27]. The observations that cinacalcet-stimulated Ca^2+^ elevation still occurred in the absence of extracellular Ca^2+^, and was not further enhanced by 4 mM extracellular Ca^2+^, add support to a CaSR-independent mechanism of Ca^2+^ elevation triggered by 30 μM cinacalcet. Of note, cinacalcet-stimulated Ca^2+^ elevation in the absence of extracellular Ca^2+^ suggests cinacalcet directly released Ca^2+^ from intracellular stores (see below). In the presence of 18 and 30 μM cinacalcet, decreases in fluorescence at both 340 and 380 nm excitation at later time points were observed, suggesting it was not only [Ca^2+^]_i_ elevation, but possibly also fura-2 leakage (Fig. 3C, D; also Fig. 5A).

Our results show that 18 μM was the threshold concentration at which cinacalcet caused membrane leakiness, mitochondrial membrane depolarization and cell growth arrest/cell death (Fig. 1, Fig. 3, Fig. 6A). Cinacalcet perturbed membrane permeability, allowing not only leakage of fura-2 and Ni^2+^ at early time points (Fig. 5), but also trypan blue uptake at later time points (Fig. 6B). In concordance, a recent report demonstrated that cinacalcet caused membrane damage in multidrug-resistant *S. aureus* [14]. How cinacalcet causes membrane permeability changes is yet to be determined. Cinacalcet’s inability to cause membrane fluidity changes at 8 min (Fig. 6C) appears to rule out the possibility that cinacalcet caused rapid membrane leakiness (Fig. 3 and Fig. 5) by enhancing membrane fluidity. Cinacalcet (10–30 μM) mildly increased membrane fluidity to a similar extent at 75 min (Fig. 6C); however, the observations that cinacalcet at 10 μM increased membrane fluidity but did not affect mitochondrial membrane potential and cell viability (Fig. 1, Fig. 6A) argue against a role of membrane fluidity changes in cinacalcet-induced suppression of cell proliferation and cell death.

Importantly, continuous Ca^2+^ influx in the presence of cinacalcet (≥18 μM) would lead to cytosolic Ca^2+^ overload, which activates multiple phospholipases, proteases and caspases [28]; cytosolic Ca^2+^ overload and hence inevitable mitochondrial Ca^2+^ overload might eventually contribute to mitochondrial membrane potential collapse and apoptotic cell death (Fig. 1). As mentioned above, the ability of cinacalcet to raise [Ca^2+^] independently of CaSR and in the absence of extracellular Ca^2+^ would imply this agent could mobilize intracellular Ca^2+^ stores in an inositol 1,4,5-trisphosphate-independent manner. Given its lipophilicity, it is possible that cinacalcet directly acted upon, in addition to endoplasmic reticulum, Ca^2+^-storing organelles such as mitochondria and lysosomes. Cinacalcet-induced leakiness in these organelles is expected to cause further damage.

There have been recent studies aiming at repurposing cinacalcet for other pharmacological applications. For example, cinacalcet has been shown in molecular docking studies to inhibit sirtuins [29]. Most of cinacalcet’s repurposed actions required higher concentrations (≥10 μM). For instance, cinacalcet (30 μM) was used to inhibit CFTR-mediated Cl^−^ flux to treat CFTR-related secretory diarrhea [11]. Similarly, cinacalcet at 30 μM was shown to inhibit cyclic nucleotide-mediated secretion in cellular and organoid models of diarrhea [30]. Due to its stimulation of phosphodiesterase breakdown of cAMP, cinacalcet was shown to suppress progression of autosomal dominant polycystic kidney disease in an animal model [12]. Inhibitory effects towards growth of multidrug-resistant Gram-positive pathogens were observed at 9–70 μM cinacalcet [13]. Cinacalcet at 10 μM was used to suppress Yes-associated protein expression to arrest proliferation of hepatocellular carcinoma [16]. We have shown in this study that cinacalcet (≥18 μM) perturbed membrane permeability and thus arrested proliferation of mouse microvascular bEND.3 EC. Hence, such ability of cinacalcet to inhibit EC growth (and thus angiogenesis) and suppress cancer cell proliferation [16–18] would render cinacalcet a potentially effective anti-tumor drug.

In conclusion, cinacalcet at ≥18 μM enhanced membrane leakiness in mouse microvascular bEND.3 EC in a manner independent of CaSR activation and membrane fluidity changes; such membrane permeability changes eventually led to cell growth arrest and cell death. Such action of cinacalcet may offer an anti-angiogenesis mechanism in suppressing tumor growth.

## Abbreviations

CaSR: Ca^2+^-sensing receptors
FCCP: Carbonyl cyanide-p-trifluoromethoxyphenylhydrazone
PAM: positive allosteric modulator
